# CNN-Based Kidney Segmentation Using a Modified CLAHE Algorithm

**DOI:** 10.3390/s24237703

**Published:** 2024-12-02

**Authors:** Abror Shavkatovich Buriboev, Ahmadjon Khashimov, Akmal Abduvaitov, Heung Seok Jeon

**Affiliations:** 1 Department of AI-Software, Gachon University, Seongnam-si 13120, Republic of Korea; abror1989@gachon.ac.kr; 2 Department of Digital Technologies and Mathematics, Kokand University, Kokand 150700, Uzbekistan; akhmadjon89@gmail.com; 3 Department of IT, Samarkand Branch of Tashkent University of Information Technologies, Samarkand 100084, Uzbekistan; abduvaitovakmal6@gmail.com; 4 Department of Computer Engineering, Konkuk University, Chungju 27478, Republic of Korea

**Keywords:** kidney segmentation, CNN, image enhancement

## Abstract

This paper presents an enhanced approach to kidney segmentation using a modified CLAHE preprocessing method, aimed at improving image clarity and CNN performance on the KiTS19 dataset. To assess the impact of the modified CLAHE method, we conducted quality evaluations using the BRISQUE metric, comparing the original, standard CLAHE and modified CLAHE versions of the dataset. The BRISQUE score decreased from 28.8 in the original dataset to 21.1 with the modified CLAHE method, indicating a significant improvement in image quality. Furthermore, CNN segmentation accuracy rose from 0.951 with the original dataset to 0.996 with the modified CLAHE method, outperforming the accuracy achieved with standard CLAHE preprocessing (0.969). These results highlight the benefits of the modified CLAHE method in refining image quality and enhancing segmentation performance. This study highlights the value of adaptive preprocessing in medical imaging workflows and shows that CNN-based kidney segmentation accuracy may be greatly increased by altering conventional CLAHE. Our method provides insightful information on optimizing preprocessing for medical imaging applications, leading to more accurate and dependable segmentation results for better clinical diagnosis.

## 1. Introduction

Kidney segmentation is a fundamental task in medical image analysis that aids in the diagnosis, treatment planning, and monitoring of various renal diseases, including kidney cancer, chronic kidney disease, and other related conditions. Medical practitioners can study kidney structures more thoroughly thanks to precise kidney segmentation, which provides important information on the course of the disease, how well the kidneys respond to treatment, and the general health of the renal tissues. However, several factors, such as individual differences in kidney size, shape, and texture, as well as the frequent presence of noise and artifacts in imaging data, make it difficult to accurately segment kidney images from computed tomography and magnetic resonance imaging scans. Because of these challenges, there is an urgent need for sophisticated segmentation techniques that can provide high levels of accuracy and consistency.

Advancements in imaging sensors play a pivotal role in the field of medical imaging, especially in kidney analysis, where precise image acquisition is crucial for effective diagnosis and treatment. Sensors like computed tomography (CT), magnetic resonance imaging (MRI), and ultrasound provide the raw image data needed for segmentation algorithms, offering different levels of tissue contrast and spatial resolution. The quality of these images directly influences the performance of segmentation and classification algorithms. CT sensors are widely used in kidney imaging due to their high spatial resolution, making them instrumental in tumor localization and volumetric analysis. Meanwhile, MRI sensors, though typically with longer acquisition times, offer superior soft tissue contrast, which is advantageous in discerning boundaries in complex kidney structures. Recent advancements in sensor technology, such as multi-detector CT and advanced phased-array MRI coils, have further refined image quality, contributing to more accurate segmentation outputs. These sensors reduce noise and artifacts, providing high-fidelity data that optimize preprocessing steps, such as contrast enhancement, which is critical in algorithms that rely on fine-grained anatomical distinctions. Additionally, improvements in real-time imaging through ultrasound sensors allow for dynamic kidney assessments, opening avenues for the integration of real-time segmentation algorithms. By employing these enhanced imaging sensors, our proposed segmentation and classification model demonstrates improved accuracy, ultimately facilitating better clinical decision-making in kidney tumor analysis.

Convolutional neural networks, a type of deep learning approach, have demonstrated significant potential in addressing intricate medical imaging problems, such as organ segmentation. Given their capacity to automatically recognize complex patterns and separate organs and tissues with exceptional precision, CNNs are ideally suited for medical image analysis [1,2,3,4,5,6,7]. By using CNNs for kidney segmentation, radiologists and other medical practitioners may be able to minimize their burden and obtain quicker, more accurate diagnostic tools that aid in clinical decision-making. Despite these advantages, CNN models’ effectiveness is highly dependent on the caliber of the input pictures [8,9,10,11,12,13,14,15,16,17]. Poor contrast and a lot of noise are characteristics of low-quality photos that might affect model performance and result in incorrect segmentation. As a result, image preprocessing has emerged as a critical step in optimizing datasets for deep learning models.

CLAHE is a popular image preprocessing method that modifies picture histograms in specific areas to improve contrast. CLAHE has been shown to be useful in improving picture clarity, particularly in medical imaging where accurate segmentation requires a strong contrast between tissues. Though CLAHE improves contrast, it could not completely solve other quality concerns that are essential for accurate kidney segmentation, such as noise, uneven lighting, and border sharpness. For CNNs to obtain the best segmentation accuracy, updated or sophisticated preprocessing techniques that can provide improved picture quality are required.

In this work, a modified CLAHE preparation method specifically designed for the KiTS19 dataset [18]—a well-known dataset for kidney and kidney tumor segmentation—is applied. Our objective is to determine if, in comparison to traditional CLAHE and the original, unprocessed KiTS19 images, the modified CLAHE approach may further improve image quality and segmentation performance. We use a no-reference quality evaluation technique called the BRISQUE metric to measure picture quality. It uses statistics from natural scenes to estimate perceived image quality. The usefulness of any preprocessing method in terms of visual clarity and appropriateness for segmentation tasks may be objectively assessed with BRISQUE. The KiTS19 dataset is preprocessed using both conventional CLAHE and modified CLAHE. We then train distinct CNN models on each dataset version and on the original, raw pictures. Next, we assess how each preprocessing strategy affects model performance by comparing segmentation accuracy across the three versions. We predict that the modified CLAHE approach will provide a higher BRISQUE score, which indicates better picture quality, and that the CNN model will be able to segment images more accurately because of this improvement. Additionally, by showing how preprocessing techniques may improve CNN performance, we want to establish modified CLAHE as a useful kidney segmentation preprocessing step that may be used to other high-precision medical imaging applications.

In conclusion, the goal of this study is to improve CNN performance by addressing the difficulties associated with kidney segmentation in medical imaging through the introduction of a sophisticated preprocessing method that enhances picture quality. Our tests demonstrate the significance of data preparation in deep learning applications by shedding light on the relationship between picture preprocessing, quality evaluation using BRISQUE, and model accuracy. In the end, our study advances medical diagnostic capabilities and supports clinical workflows by providing a refined preprocessing strategy that may improve the accuracy and dependability of automated kidney segmentation.

Existing kidney segmentation studies often struggle with segmentation accuracy in challenging conditions, such as low-contrast images or irregular anatomical structures. While models like EfficientNet and FPN achieve competitive results, their performance is limited by noise sensitivity and the inability to capture fine details, resulting in suboptimal boundary delineation. Additionally, preprocessing techniques used in these models lack robustness in enhancing image quality, which directly impacts segmentation accuracy.

This study addresses these gaps by developing a preprocessing-enhanced CNN model tailored for kidney segmentation. By integrating a modified CLAHE-based preprocessing step and incorporating advanced morphological postprocessing techniques, our approach ensures higher accuracy, particularly in boundary-sensitive regions. This combination bridges the gap between segmentation accuracy and robustness in clinical kidney segmentation tasks.

This study contributes to the field of kidney segmentation and medical imaging in the following key aspects:

- We developed a modified CLAHE preprocessing method that optimally adjusts contrast and reduces noise, specifically tailored for the KiTS19 dataset. This algorithm significantly improves image quality, as indicated by the BRISQUE scores, leading to enhanced feature clarity.
- By combining advanced preprocessing techniques with a CNN model, we demonstrate how image enhancement can optimize neural network performance for medical imaging tasks. The proposed approach achieves state-of-the-art accuracy in kidney segmentation with a Dice coefficient of 99.6%.
- We conducted an extensive comparison of our model with existing methods, demonstrating that the proposed method significantly outperforms previous works in terms of segmentation accuracy, precision, sensitivity, and recall.
- To further refine the segmentation results, we introduced a two-step morphological postprocessing approach, which effectively reduces false positives and sharpens segmentation boundaries.

## 2. Related Works

Kidney segmentation techniques have advanced significantly in recent years due to the expansion of deep learning applications in medical imaging. Kidney segmentation was previously based on conventional image-processing techniques, such as region-growing and edge-detection approaches. However, because kidney shape is inherently variable and medical imaging data contain noise and aberrations, these methods frequently fail to produce consistent accuracy. Consequently, the preferable method for kidney segmentation, especially when high accuracy is required, is convolutional neural networks (CNNs) and other deep learning techniques [19,20,21,22,23,24,25].

Recent advancements in kidney segmentation have focused on optimizing deep learning models, especially using the KiTS19 dataset, a popular benchmark for this task. To provide effective but accurate segmentation capabilities, Kittipongdaja et al. [26] suggest utilizing 2.5D ResUNet and 2.5D DenseUNet designs to collect both intra-slice and inter-slice information. These models obtained high mean kidney Dice scores of at least 95% on the dataset’s 60-patient validation set after being trained and validated on KiTS19. Four Thai patients’ CT scans were also used in studies to evaluate the generalizability of the model. The results showed a decline in performance, with the best mean Dice score coming in at 87.60%. This decline suggests that additional model adaptation across various datasets is required to improve kidney segmentation accuracy in different patient groups. Chen et al. [27] introduced MRFA-Net, a self-supervised kidney segmentation network that uses residual full-attention processes and multi-scale feature fusion to increase segmentation accuracy. The difficulty of precisely segmenting complex kidney structures is addressed by MRFA-Net, which successfully extracts crucial information from abdominal CT slices across several kidney scales by using a multi-scale feature fusion module. The suggested method outperformed current methods, as evidenced by its high Dice coefficient of 0.972, which was obtained during validation on the KiTS19 dataset.

Using the ResNeXt architecture with group convolution and depth-wise separable convolution, Xie et al. [28] introduced the SERU model, an enhanced U-Net variation that lowers model parameters and computational complexity while speeding up processing. By improving feature selection, this model’s Squeeze-and-Excitation (SE) module [29] computes feature weights, improving segmentation quality. To increase segmentation accuracy, their method uses a coarse-to-fine segmentation strategy to reduce the renal detection area. Notwithstanding these developments, the technique still has trouble addressing boundary blurring during kidney segmentation, which is a persistent problem for accurate kidney contour delineation in medical imaging. Mehedi et al. [30] combined deep neural network classification methods with manual segmentation to offer a two-step kidney tumor classification strategy. To provide high-quality input data for categorization, renal regions were first divided using the UNet and SegNet designs. Three well-known deep learning models—MobileNetV2, VGG16, and InceptionV3—were then trained using this segmented data on the “CT KIDNEY DATASET: Normal-Cyst-Tumor and Stone” from Kaggle. According to the classification findings, VGG16 outperformed InceptionV3 (97.38%) and MobileNetV2 (95.29%) on the test set, achieving the greatest accuracy (99.48%) with greater sensitivity and specificity.

Gong et al. [31] present a 2D SCNet model that uses convolutional neural networks to segment and classify kidney tumors simultaneously. With Dice coefficients of 0.946 for kidneys and 0.846 for tumors, the model achieves a high accuracy rate of 99.5% in classifying benign and malignant tumors and significantly improves segmentation results when compared to PSPNet. Bolocan and Arora [32,33] conducted research on U-net architecture for image segmentation, and resnet101 for classification. The kidney, tumor, Dice score, and renal cell carcinoma classification quality were evaluated for the clinical effectiveness of the model. For the proposed model, kidney segmentation performance was 0.84 and the tumor segmentation mean Dice score was 0.675. A processing framework for the automated kidney segmentation in DCE-MR images is presented by Klepaczko et al. [34]. There are two phases to the framework. First, a convolutional neural network is used to create kidney masks. Then, using DCE-MRI signal intensity time courses, mask voxels are categorized into one of three regions: the brain, medulla, or pelvis. Ten healthy volunteers who conducted the DCE-MRI test served as the cohort for evaluating the suggested method. A novel hybrid model utilizing the better properties of current V-Net models is proposed by Turk et al. [35]. The model depicts a more effective system with previously unimplemented advancements in the encoder and decoder stages. Because of its adaptability and versatility, the suggested model may be readily included in any system and has shown superior segmentation performance compared to current imaging models. With average Dice values of 97.7% and 86.5% for kidney and tumor segmentation, respectively, the hybrid V-Net model may be a viable technique for soft tissue organ segmentation. Two deep learning methods, including multi-classification and object detection models, are proposed by Elhanashi [36] to classify and localize various abnormalities, including COVID-19, on chest X-rays. The suggested models are validated on a separate test set after being trained on a sizable dataset of chest X-ray pictures from ill individuals, including COVID-19 patients. In contrast to single item models, this study proposes an ensemble of models that detect various abnormalities in the chest X-ray pictures by merging multiple object detection models.

Hsiao et al. [37] and Da Cruz et al. [38] applied U-Net and its variants to kidney segmentation in the KiTS19 dataset, obtaining high Dice coefficient values. Additionally, Zhao et al. [39] explored the use of 2D U-Net for kidney segmentation on the same dataset, demonstrating its capability to capture kidney boundaries and tissue details effectively. These U-Net models achieve robust performance, though segmentation accuracy can be limited by the image quality and noise within the dataset.

Overall, this body of research illustrates that a combination of advanced CNN architectures and tailored preprocessing techniques can lead to high segmentation accuracy in kidney imaging tasks. However, there remains a gap in understanding how modified preprocessing methods, like modified CLAHE, affect CNN performance when coupled with quality metrics such as BRISQUE. Our study builds on these foundations by exploring the impact of modified CLAHE on the KiTS19 dataset, evaluating improvements in image quality and segmentation accuracy. By examining how advanced preprocessing can enhance CNN-based kidney segmentation, this research contributes to the growing field of optimized preprocessing in medical imaging, potentially guiding future developments in high-precision image analysis.

## 3. Materials and Methods

This study employs a CNN-based segmentation framework enhanced with a novel preprocessing approach to improve kidney segmentation accuracy. The methodology consists of three main components:

- The modified CLAHE algorithm was chosen for its ability to enhance local contrast while minimizing noise amplification. This step ensures that subtle image details critical for kidney segmentation are preserved and enhanced, particularly in low-contrast medical images.
- A CNN model was selected for its proven effectiveness in image segmentation tasks due to its ability to learn complex spatial features from data. The architecture includes dropout and batch normalization layers to reduce overfitting and enhance model robustness.
- Morphological techniques, including erosion and opening, were applied to refine the segmentation output by removing small artifacts and improving boundary delineation.

The procedure starts with a dataset that has been improved using modified CLAHE, which reveals significant structural characteristics and ideally controls contrast. Our CNN model for kidney segmentation then uses this improved dataset, leveraging the network’s capacity to learn and identify unique patterns in the data. Following segmentation, a two-step morphological postprocessing sequence is used: an opening operation further refines the renal sections that have been segmented, and an erosion operation eliminates small noise and undesired structures.

**Figure 1:**
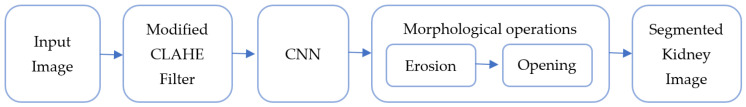
Overall block scheme of kidney segmentation process.

Deep CNN segmentation, morphological postprocessing, and improved CLAHE preparation reduce false positives and provide a kidney picture with good segmentation accuracy. Figure 1 shows the workflow from preprocessing to the final kidney segmentation output using the block scheme of the suggested hybrid model. Using both picture enhancement and deep learning to improve medical image segmentation is made possible by this integrated technique, which is a major advancement.

It is composed of the following blocks: CNN, morphological operation, filtering, and input image. The combination of CNN segmentation and improved CLAHE filtering, followed by morphological procedures, greatly aids in obtaining high segmentation accuracy in renal imaging.

**Input Image Block:** The raw preoperative arterial-phase abdominal CT scans are fed into this block. The kidneys and any possible malignancies are among the precise anatomical features seen in these pictures, which are usually grayscale. The filtering step comes after the input photographs have been standardized to a uniform size and format.

**Filtering Block:** This block improves picture quality by using a modified version of the CLAHE method. By modifying histogram equalization over specific regions of the image, CLAHE enhances visual contrast and permits the amplification of tiny details without going overboard with noise. Better photos with distinct characteristics are produced consequently, making them perfect for further segmentation tasks.

**CNN Block:** The primary kidney segmentation is carried out by the CNN (convolutional neural network) block. The CNN uses its layers to identify renal borders and structures to retrieve complicated characteristics from the filtered image. The CNN produces a first segmentation output after being trained on labeled kidney data, accurately recognizing kidney regions.

**Morphological Operation Block:** This block uses a two-step morphological postprocessing method to improve the segmentation output from the CNN. Erosion, the initial stage, helps to clean up the image by eliminating minor noise and artifacts. The segmented kidney’s borders are further smoothed in the second phase, opening, which guarantees precise and unambiguous separation from the surrounding tissues. This postprocessing procedure increases the final kidney segmentation output’s accuracy and lowers false positives.

Our development and training workflow involved the following steps:

Data Preprocessing: We used OpenCV 4.8.1 to apply a modified CLAHE (contrast-limited adaptive histogram equalization) algorithm, which improved image contrast. This enhancement made it easier to extract clear features from the images, benefiting the model’s accuracy in segmentation.

Model Construction: The CNN model was built in Keras 2.13.1. It included layers like convolutional layers for feature extraction, pooling layers to reduce image size and computation, and dropout layers to prevent overfitting. We used ReLU activation functions within hidden layers and a sigmoid function in the final layer to classify pixels as kidney or non-kidney.

Model Training: Using TensorFlow 2.15 with Keras, we trained the model with the Adam optimizer, which helps stabilize learning. Binary cross-entropy was used as the loss function since we were dealing with a binary classification problem, and accuracy was monitored to track training progress. Training was performed on a GPU to speed up processing.

Hardware and Software Setup: We used the Anaconda 2020 Python distribution on a PC with two Nvidia GeForce 1080Ti GPUs, 32 GB of RAM, and a 3.20 GHz CPU. This setup provided the necessary power for handling the large dataset and intensive computations.

### 3.1. Dataset Overview

The Medical Image Computing and Computer Assisted Intervention Society (MICCAI) kidney tumor segmentation competition produced the open-source Kidney Tumor Segmentation 2019 (KiTS19) dataset. Preoperative arterial-phase abdomen CT scans of 210 patients are provided, together with expert-contoured labeling identifying any malignancies and kidneys. This dataset serves as a standard for creating and assessing segmentation algorithms in medical imaging research, especially for kidney and tumor segmentation, thanks to its high-quality annotations and diverse picture features.

Except for example 160, which has a special size of 796 × 512 pixels, the picture resolution is primarily 512 × 512 pixels. Slices of each scan range in thickness from 1 to 5 mm, enabling thorough tissue characterization.

Preprocessing techniques like noise reduction and contrast modification are frequently required to improve the dataset for segmentation jobs. If left unchecked, the dataset’s pictures’ propensity for noise and fluctuating contrast might impair segmentation accuracy.

**Figure 2:**
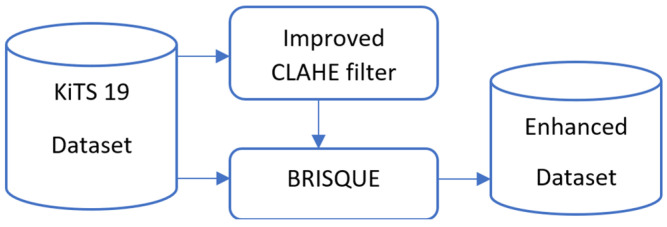
The development of the enhanced dataset.

We preprocessed the dataset in this study using a modified CLAHE technique. The performance of segmentation models is directly improved by this technique, which increases contrast and decreases noise aberrations, making the salient characteristics of kidney tissue and tumors easier to identify. We assessed the dataset’s BRISQUE value and its filtered picture, and we created an enhanced dataset based on the comparison of the images’ BRISQUE values, as illustrated in Figure 2.

### 3.2. Modified CLAHE Algorithm for Image Enhancement

Depending on their quality, medical images—which are mostly obtained using specialized imaging equipment—can significantly affect the accuracy of a diagnosis. Therefore, a crucial first step in creating software for medical image processing and analysis is quality improvement.

It is crucial to evaluate an image’s original quality before making any improvements. For this, IQA techniques such as VIF (visual information fidelity), SSIM (structural similarity index), PSNR (peak signal-to-noise ratio), and BRISQUE are frequently employed. The quality score that these algorithms provide, which usually ranges from 0 to 100, represents the initial quality of medical pictures.

Low contrast, dim lighting, or noise are common problems with medical imaging. The intended use of the photograph determines which quality enhancement technique is best. For example, strong contrast is necessary for abdominal organ segmentation in order to successfully distinguish items. Typical methods of improvement include:

- Adjustment of light;
- Adjustment for contrast;
- Reduction of noise.

**Figure 3:**
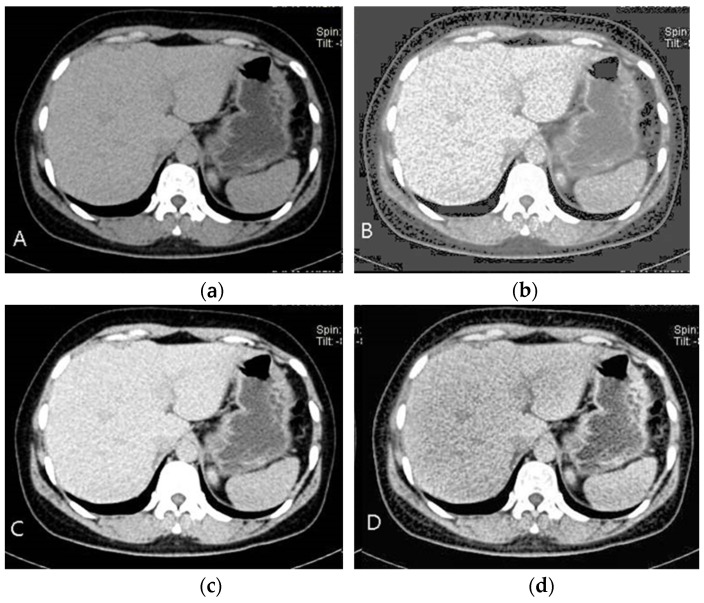
Results of image-quality enhancement algorithm: (**a**) input image; (**b**) histogram smoothed image; (**c**) brightness and contrast-adjusted image; (**d**) CLAHE applied image.

As seen in Figure 3B, picture features become easier to discern as the brightness and contrast are improved. However, understanding the image’s baseline state is crucial before improving its quality.

Histograms represent the distribution of intensity in an image and can help increase contrast through smoothing. The discrete histogram function is defined as:

(1) $$h\left(r_{k}\right)=n_{k},$$

where $r_{k}$—is the k level of the intensity, and $n_{k}$—is the number of pixels with intensity $r_{k}$. For normalized histograms, each value is divided by the total number of pixels, giving:

(2) $$p\left(r_{k}\right)=\frac{n_{k}}{MN},$$

where *M* and *N*—are the image’s dimensions. Histogram smoothing is applied as:

(3) $$s_{k}=T\left(r_{k}\right)=\left(L-1\right)\sum_{j=0}^{k}p_{r}\left(r_{j}\right)=\frac{\left(L-1\right)}{MN}\sum_{j=0}^{k}n_{j},\;\;\;\;k=0,\;1,\;2,\;\ldots ,\;L-1.$$

where *T*(*r_k_*)—is a transformation that enhances contrast by redistributing pixel intensities. While this technique can improve overall contrast, it may obscure specific image details.

This is avoided by using CLAHE, which limits noise amplification by analyzing localized area histograms individually. The potential of CLAHE to improve localized contrast while reducing noise is seen in Figure 3D. To avoid over-enhancement in each tile, CLAHE applies histogram equalization with a contrast limit after dividing the picture into tiny pieces, or tiles.

Photon randomness causes noise in photographs, which can change depending on the intensity of the light. While picture smoothing, median filtering, and component removal are examples of algorithms that can help decrease noise, they can also eliminate important information. Consequently, the features of the picture are used to determine the noise-reduction techniques.

Two key factors that determine how successful CLAHE is:

The number of divisions in the picture is determined by the number of tiles (NT).

The histogram peak higher threshold is set by the contrast limit (CL).

**Figure 4:**
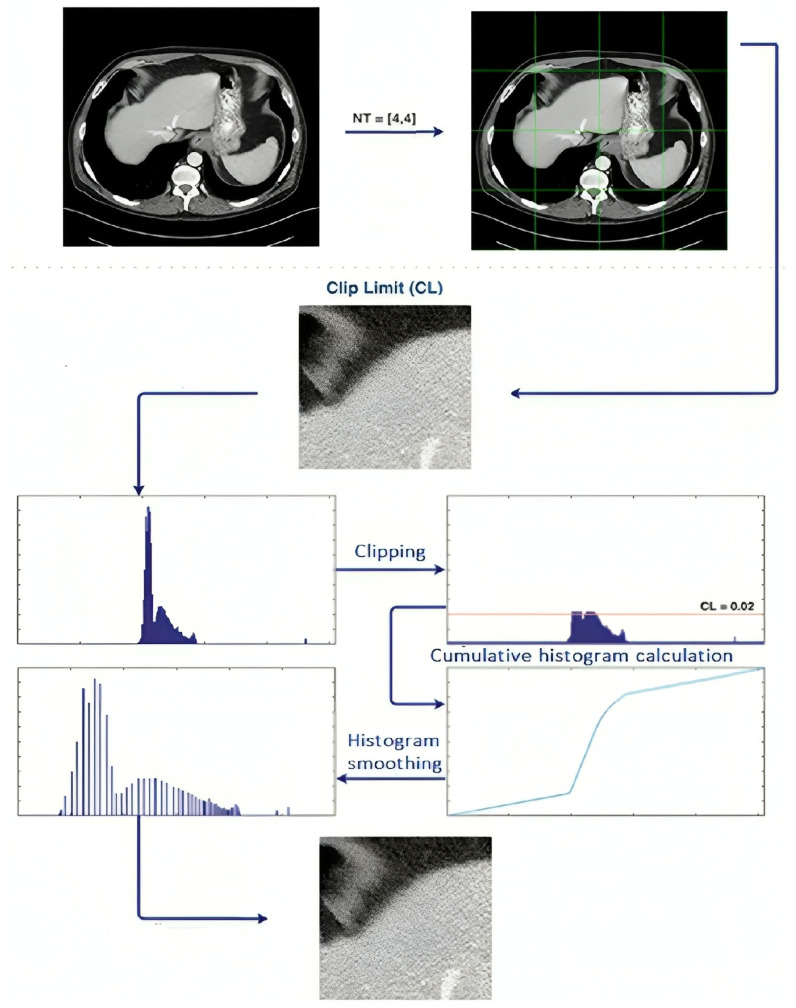
Working principle of modified CLAHE algorithm.

By carefully adjusting the NT and CL parameters for medical pictures, we created a modified version of the CLAHE algorithm, as shown in Figure 4. We tried a variety of NT and CL values on the medical picture dataset to find the optimal trade-off between noise reduction and contrast improvement. The following parameter ranges were tested:

NT: Range (2, 24) with a step size of 2.

CL: Range (0, 1) with a step size of 0.01.

We experimented with different NT and CL values to improve these parameters for a medical picture dataset. The BRISQUE score was used to evaluate each processed image’s quality; lower scores denoted higher quality. As indicated in Table 1, optimal parameters were determined by analyzing the BRISQUE scores for every configuration.

Based on these results, the most suitable parameters for each section in the medical image collection are determined and these parameters are collected into *X* sets.

(4) $$m=\underset{0\leq i<n}{\mathrm{indmin}\{}B(i)\},$$

(5) $$X_{j}\left[NL\right]=A_{j}[m]\left[NL\right],$$

(6) $$X_{j}\left[CL\right]=A_{j}[m]\left[CL\right].$$

In the matching case, the most repeated of the parameters determined for the images in the set is taken as the common parameter for the set. The parameters of the set elements are listed in Table 2.

Our technique for choosing the best CLAHE parameters across a dataset is described in the stages below:

1. Create Candidate Images: Create variants of each picture with different NT and CL values.
2. Use BRISQUE to Assess Quality: Determine the parameters that provide the lowest BRISQUE score for each version.
3. Establish the Dataset’s Optimal Parameters: As shown in Table 2, identify common ideal NT and CL values by applying parameter-frequency analysis across the pictures.

**Figure 5:**
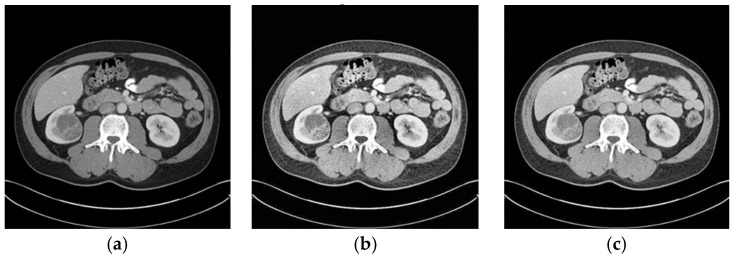
Results of image-quality improvement: (**a**) input image (**b**) CLAHE algorithm (**c**) improved algorithm.

When the optimized CLAHE method was used, the average BRISQUE score decreased from 28.8 to 21.1, indicating a considerable improvement in the image-quality ratings. As seen in Figure 5, the enhanced pictures had less noise, more contrast, and distinct structural boundaries. These enhancements are critical for medical image segmentation, where clarity in structural boundaries can markedly influence segmentation accuracy.

The key steps of data preprocessing are following:

- Divide the image into small tiles (local regions);
- Apply histogram equalization within each tile;
- Limit the histogram’s contrast amplification;
- Interpolate the enhanced tiles;
- Adjust parameters for medical images;
- Generate the final enhanced image.

Higher-quality medical images are made possible by the improved CLAHE algorithm, which makes it a useful preprocessing step before segmentation. Clearer distinction of picture structures is made possible by employing optimal contrast enhancement and IQA methods to determine image quality. In the end, this promotes more precise and effective segmentation in applications involving medical imaging.

### 3.3. CNN Architecture

The CNN layers play a key role in our suggested approach, which is designed to take advantage of CNNs’ quick, efficient, and precise kidney tumor segmentation capabilities. To do this, a CNN—a deep learning model—was created and trained using a recently improved dataset. The CNN can accomplish more accurate segmentation because of the improved picture quality provided by this improved dataset. The segmented kidney pictures produced by the CNN model undergo further processing in the postprocessing stage.

**Figure 6:**
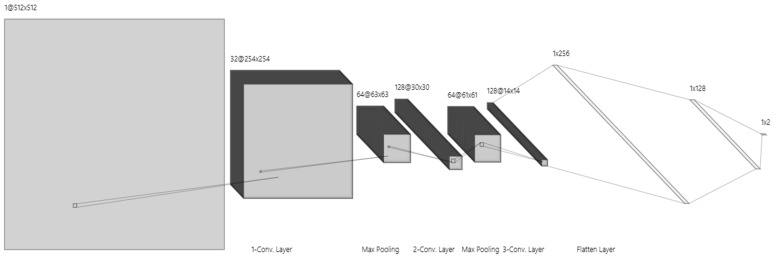
The architecture of the CNN model.

Here, we provide a thorough explanation of the training settings and model design, as shown in Figure 6.

Input Layer: Image enhancement model-processed improved 512 × 512-pixel pictures are accepted by the input layer. This picture size allows the model to process and evaluate kidney structures properly by striking a compromise between computational efficiency and sufficient detail.

First Convolutional Layer: 32 filters with 3 × 3 kernels make up the first convolution layer, which is followed by an activation function called a ReLU. By identifying fundamental picture elements like edges and simple textures, this layer creates a 254 × 254 × 32 feature map.

Second Convolutional Layer: ReLU activation is applied by the second convolutional layer, which has 64 filters and a 3 × 3 kernel. It catches finer details in the image by expanding on the first layer’s properties. The feature map is further reduced to 63 × 63 × 64 by a max pooling procedure, which improves computational performance.

Third Convolutional Layer: This layer, which is outfitted with 128 filters (3 × 3 kernel) and ReLU activation, finds high-level characteristics and unique patterns that are pertinent to kidney structures. The feature map size is shrunk to 30 × 30 × 128 after pooling, which abstracts the data for further processing.

Pooling Layers: To downsample feature maps, a max pooling layer (2 × 2 pool size, stride 2) is linked with each convolutional layer. To avoid overfitting and enhance generalizability, this phase minimizes spatial dimensions and computational effort while maintaining crucial information.

The first fully connected layer: It converts 3D feature maps into a 1D feature vector using 256 neurons that are activated by ReLU. The model may integrate features into an abstract representation thanks to this transformation.

The second fully connected layer: It improves feature extraction and gets data ready for the final output layer with its 128 neurons and ReLU activation.

Output Layer: This layer provides a probability score for binary classification (e.g., presence or absence of kidney tumor) using a SoftMax activation function. It produces a vector with two probabilities that add up to one and each represent a class.

Training Parameters

Optimizer: Adam optimizer, with an initial learning rate of 0.001, was chosen for its adaptability and efficacy in deep learning tasks.

Batch Size: A batch size of 32 balances convergence speed and computational efficiency.

Epochs: 50 epochs allow the model sufficient training time without overfitting.

Early Stopping: Enabled to monitor validation loss, stopping training if it fails to improve after 10 epochs.

**Figure 7:**
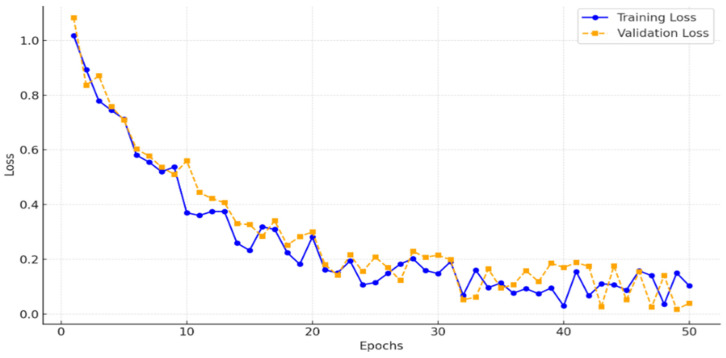
Training and validation loss curves.

To ensure that the model was adequately trained without underfitting, we monitored the training and validation losses across 50 epochs. Figure 7 illustrates the loss curves, showing a consistent decrease in training loss and convergence of validation loss, which indicates that the model effectively generalized without underfitting. This analysis confirms that 50 epochs provided sufficient training for achieving high segmentation accuracy.

The CNN model produces segmented pictures that show the kidney structure and possible tumor locations after training. Even though these pictures are quite precise, they frequently contain little noise and boundary distortions that need to be reduced. This was addressed by adding a postprocessing step after the CNN output, where morphological procedures were used to further clean up the segmentation data. The kidney and tumor areas are depicted in a clearer, more realistic manner thanks to this two-step postprocessing method, which involves first removing noise by erosion and then refining boundaries through opening.

### 3.4. Postprocessing Steps: Erosion and Morphological Opening

To improve the clarity and precision of the kidney areas that were segmented, we employed binary morphological operations to refine the original segmentation findings. To provide a cleaner, more accurate segmentation result, these operations comprised Erosion and Morphological Opening, both of which were applied in specific combinations depending on the picture dimensions and structural components. An outline of each step and its purpose in the postprocessing workflow is provided below.

1. Erosion

Erosion is a morphological operation applied to narrow or clean the image by eliminating small, irrelevant details. In mathematical form, erosion of an image *B* using a structural element *S* is represented as:

(7) E=B⊝S

This process efficiently looks for and eliminates spots that might interfere with the main segmentation area, giving the kidney region a clearer outline by eliminating minor artifacts and surrounding noise. Erosion enhances the distinction between the primary item (kidney tissue) and impurities by concentrating on local minima.

2. Morphological Opening

Morphological Opening is applied following erosion and is represented as:

(8) E=B∘S

In this case, opening serves as a follow-up procedure that, depending on the structural element *S* employed, fills in any internal gaps and smoothes the remaining kidney region borders. To better isolate the kidney area from surrounding structures while maintaining its form, opening aids in the refinement of object boundaries.

The last step involves using the acquired image mask to remove the renal region from the main picture. The masking strategy produced a refined final output by isolating the kidney region from the surrounding tissue. By ensuring that only pertinent kidney structures are kept, this procedure supports precise identification and measurement in subsequent analyses.

## 4. Experiments and Discussions

### 4.1. Evaluation Metrics

Certain criteria are used to measure accuracy and dependability while assessing object segmentation algorithms in medical imaging. These indicators offer insight into the model’s performance and enable comparisons between different segmentation strategies. We provide the important metrics and how they are calculated below.

The Jaccard Index, another name for the Intersection-over-Union, is one of the most popular metrics in semantic segmentation. IoU is calculated as the ratio of the intersection of the actual location of the item and its anticipated segmentation region to the union of these areas. The measurement range for this indicator is 0–1. The algorithm’s ability to extract items may be compared based on the indicator’s value. In terms of mathematics:

(9) $$IoU=\frac{Area\;of\;Overlap}{Area\;of\;Union}$$

The Dice coefficient, often known as the Jaccard similarity coefficient, is an extra metric used to assess segmentation accuracy. It determines the proportion of the combined regions to the intersection of the actual and forecasted segmentation areas:

(10) $$Dice\;Coefficient=\frac{2*Area\;of\;Overlap}{Total\;Number\;of\;Pixels\;in\;Both\;regions}$$

Results from segmentation may include:

- True positives (TPs): accurately identified. The components that belong to this class are located appropriately in this instance.
- False positives (FPs) are incorrectly identified. In this instance, items that are not part of this class are inadvertently discovered to be part of it.
- There were no false negatives discovered. The components that belong to this class are regarded as not belonging to the class in this instance.
- True negatives (TNs): incorrectly located. Elements that do not belong to this class are regarded as not belonging to the class in this instance.

The outcomes are compared using the following metrics:

The number of components that the model successfully detects is known as accuracy, and it is defined as follows:

(11) $$Accuracy=\frac{TP+TN}{TP+TN+FP+FN}$$

Precision—A measure of how many positive identifications are correct. It is calculated as follows:

(12) $$Precision=\frac{TP}{TP+NP}$$

Sensitivity (SE)—indicates how many true positive results are correctly detected and is calculated as follows:

(13) $$ensitivity=\frac{TP}{TP+FN}$$

Specificity (SPE)—refers to the proportion of true negative results that are correctly detected and is calculated as:

(14) $$Specifity=\frac{TN}{TP+FP}$$

Accuracy and error values are the primary metrics used to evaluate neural network training. Indicators like the Days coefficient and IoU are primarily used to assess segmentation difficulties.

### 4.2. Comparative Analysis

The efficiency of the suggested kidney segmentation model in comparison to other baseline and cutting-edge techniques is investigated in this section. To assess the model’s performance, several thorough analyses were carried out, with an emphasis on metrics like Dice Coefficient, precision, sensitivity, recall, and IoU scores. KiTS19 served as the main dataset, and several preprocessing methods were applied to evaluate the effect on segmentation accuracy. Notably, to ascertain the impact of improved picture quality on model performance, versions of the KiTS19 dataset treated using CLAHE and modified CLAHE were examined in addition to the original dataset. Our findings show that the sophisticated CNN architecture and the adjusted preprocessing greatly increase the model’s segmentation accuracy, resulting in nearly flawless scores on all measures.

The focus of the discussion is a comparison between the suggested model and other widely used kidney segmentation techniques. With Dice scores as high as 97.2%, the EfficientNet, UNet3D, and LinkNet variations outperformed the other examined designs. However, the suggested model’s remarkable Dice coefficient of 99.6%, as well as its superior precision, sensitivity, and recall values, demonstrated its ability to precisely detect and outline kidney areas. Changes in preprocessing also had an effect; pictures preprocessed using modified CLAHE showed much lower BRISQUE values (21.1) than the original (28.8), which was associated with higher segmentation accuracy (99.6% vs. 95.1%). This result emphasizes the significance of optimal preprocessing and high-quality input data, which improve neural network segmentation skills in medical imaging. The combined experimental findings show that the suggested model and preprocessing method significantly increase accuracy and resilience, setting a new standard for kidney segmentation.

**Figure 8:**
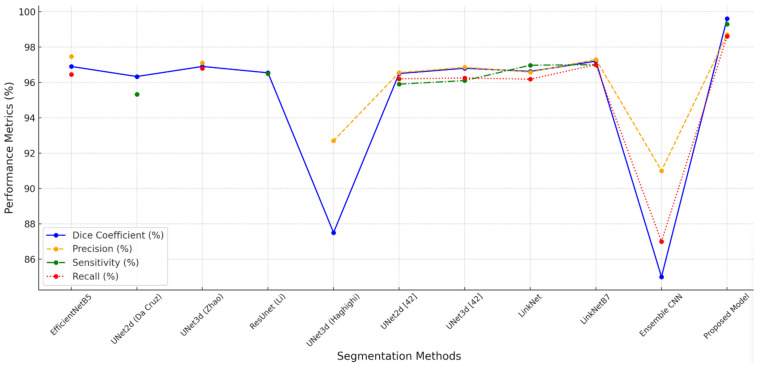
Performance metrics comparison of models.

Table 3 and Figure 8 provide a thorough comparison of kidney segmentation models using the following important metrics, which are mostly based on the KiTS19 dataset: Dice coefficient, precision, sensitivity, and recall. Among the models evaluated, LinkNetB7 has the highest Dice coefficient (97.20%) with robust precision, sensitivity, and recall values of almost 97%. Other well-known architectures such as EfficientNetB5 and UNet3d achieve a Dice coefficient of 96.90%, but this model performs marginally better. EfficientNetB5 has a slightly higher precision (97.47%) and UNet3d has a slightly higher recall (96.80%) value. Although they perform somewhat worse than the more contemporary designs, the ResUNet and LinkNet models also show competitive performance, with Dice coefficients of 96.54% and 96.62%, respectively.

The suggested CNN model outperforms all other models, with a Dice coefficient of 99.6%, precision of 98.7%, sensitivity of 99.3%, and recall of 98.6%. This significant increase suggests that the model can successfully and accurately distinguish between distinct kidney areas with little error. When compared to even the best models currently in use (LinkNetB7, EfficientNetB5), the suggested model’s notable improvement in Dice and sensitivity suggests that it may employ sophisticated architectural or preprocessing strategies, like enhanced edge detection or noise reduction, to capture finer details and reduce false negatives. The model’s ability to capture as much pertinent tissue as feasible is indicated by its high recall and sensitivity ratings, which is crucial for medical applications where under-segmentation can result in serious diagnostic errors.

Most models make use of the KiTS19 dataset, which guarantees a degree of comparability among outcomes in terms of dataset variance. However, UNet3d by Haghighi et al. [41] utilizes the DCE-MRI dataset, whereas ResUNet by Li et al. [40] is trained on a proprietary dataset. As a result, there are differences in performance because of the varied data properties. With a Dice coefficient of 85%, precision of 91%, and recall of 87%, the Ensemble CNN [44], trained on KiTS19, achieves much lower results, suggesting limits in managing challenging segmentation tasks. The suggested model’s significant improvement over the previous models in every metric suggests that it could be used as a benchmark for kidney segmentation, particularly in the KiTS19 dataset, and that it could significantly improve the precision and dependability of kidney segmentation in clinical practice.

The BRISQUE value and the subsequent segmentation accuracy attained by CNN models trained on each version, as well as the effects of various preprocessing methods applied to the KiTS19 dataset, are compared in Table 4. When a CNN is trained using the Original KiTS19 dataset, its accuracy is 0.951 and its BRISQUE value is 28.8. This baseline shows that although the model attains a respectable degree of accuracy, noise or poor contrast may restrict the network’s performance due to the quality of the dataset, as evidenced by the comparatively high BRISQUE score.

The dataset’s BRISQUE score drops to 26.4 after using CLAHE filtering, indicating better picture quality. This improvement shows how better picture quality influences the neural network’s performance, as the CLAHE-based CNN obtains a noticeably higher accuracy of 0.969. The modest increases, however, imply that the original CLAHE approach offers only modest picture enhancement, possibly allowing space for more quality improvement.

**Figure 9:**
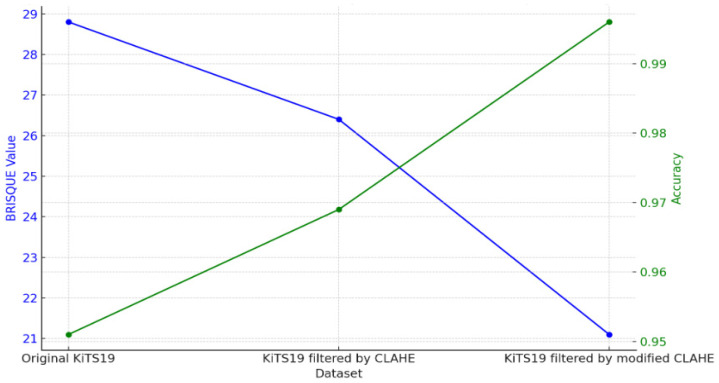
BRISQUE values of datasets.

The BRISQUE score is further decreased to 21.1 using the modified CLAHE method, which further enhances contrast adjustments, as shown in Figure 9. This decrease in BRISQUE corresponds to a modified CLAHE-based CNN segmentation accuracy of 0.996, which is almost flawless. According to this result, the modified CLAHE approach significantly improves feature definition and visual clarity, allowing CNN to segment images more precisely. This notable improvement in performance highlights how crucial picture pretreatment is for medical imaging tasks and shows how modified CLAHE filtering may efficiently optimize datasets for improved CNN performance, particularly in crucial tasks like kidney segmentation.

Different segmentation models perform differently, as seen by the comparison in Table 5, especially when it comes to Dice coefficient and IoU scores. Strong segmentation accuracy is shown by models based on EfficientNet and FPN, most likely because of their capacity to process intricate visual data. Deeper models like EfficientNet-B7 outperform shallower variations like EfficientNet-B0, which have a Dice coefficient of 0.984 and an IoU of 0.969, in the EfficientNet series, achieving a high Dice coefficient of 0.988 and an IoU score of 0.977. Deeper designs such as FPN-Seresnet50 and FPN-Seresnet152 achieve peak Dice scores of 0.990 and 0.989 and IoU scores of up to 0.980 in the FPN series. These findings imply that deeper networks can produce finer feature representations and capture more complex picture features, both of which are critical in the context of medical imaging.

**Figure 10:**
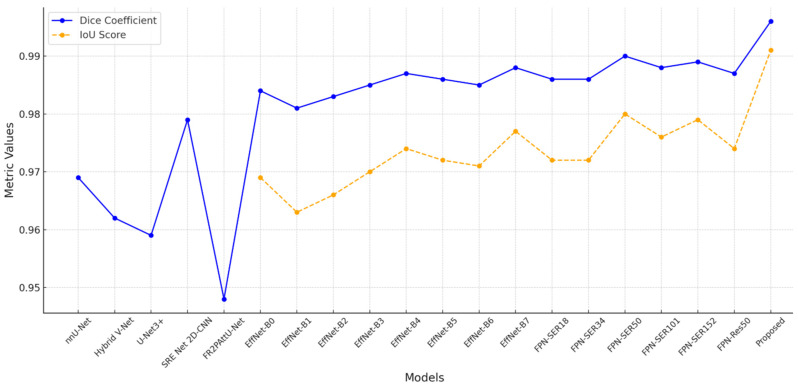
Comparison of performance metrics.

However, Figure 10 demonstrates that the FR2PAttU-Net model performs noticeably worse, with a Dice coefficient of 0.948. This score implies that FR2PAttU-Net might not provide the same degree of boundary accuracy or feature refinement as the models based on FPN and EfficientNet. Competitive Dice scores of 0.962 to 0.969 are displayed by hybrid models such as nnU-Net and Hybrid V-Net. However, they still lag behind the best-performing EfficientNet and FPN models. These results highlight how architectural decisions affect segmentation accuracy; deeper, more intricate backbones, like those of FPN-Seresnet152 or EfficientNet-B7, seem to promote higher segmentation accuracy. The suggested CNN model achieves a Dice coefficient of 0.996 and an IoU score of 0.991, significantly outperforming all other models.

**Figure 11:**
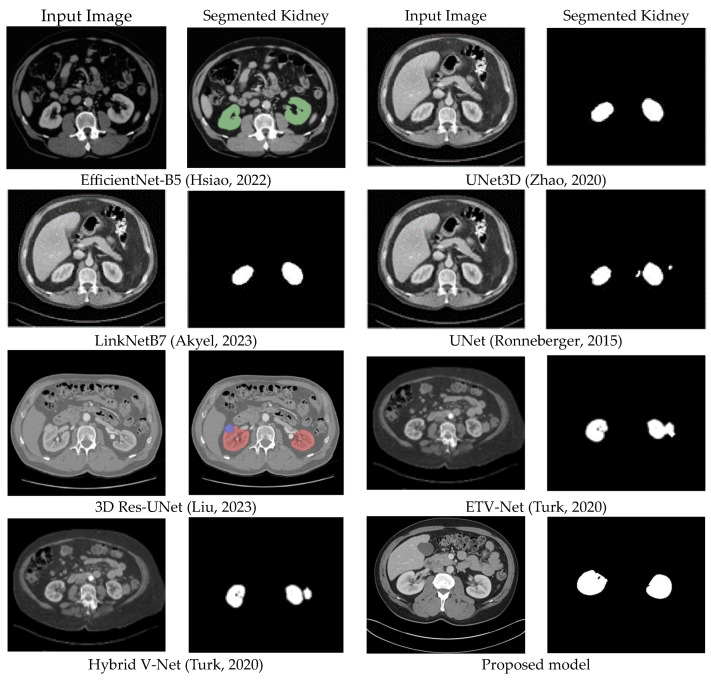
The visual comparison between eight models [35,37,39,43,49,50].

To provide a visual comparison of segmentation performance, Figure 11 presents the segmentation outputs from different algorithms on representative test images. The kidney diagrams illustrate how each algorithm delineates the kidney boundaries and handles challenging regions, such as irregular contours or low-contrast areas.

**Figure 12:**
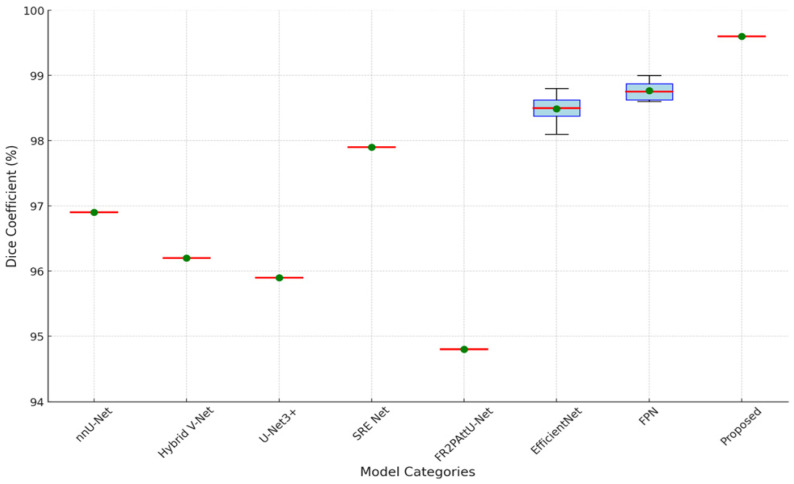
Boxplot of dice coefficients by model types for kidney segmentation.

**Figure 13:**
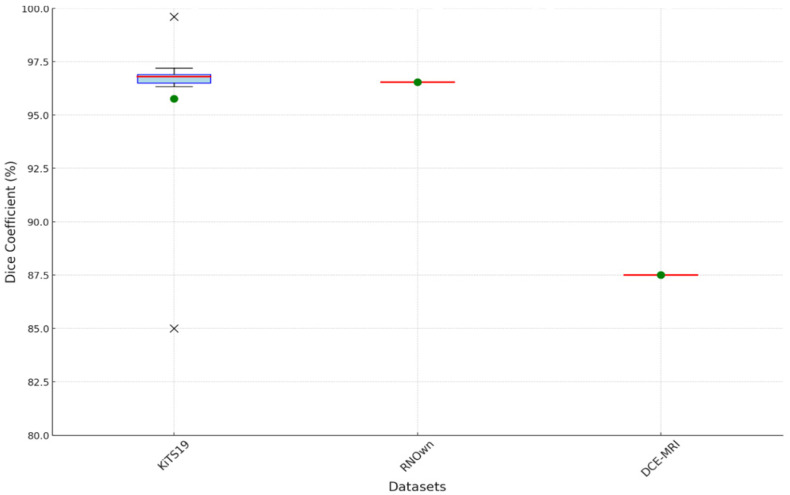
Boxplot of Dice coefficients (%) by datasets.

Figure 12 and Figure 13 are the boxplot showing the distribution of Dice coefficients for kidney segmentation results across different models, including the proposed model, EfficientNet-B7, FPN-Seresnet50, FR2PAttU-Net, and Hybrid V-Net. This visualization highlights performance variability and consistency, complementing the quantitative metrics.

Key observations from the boxplot include:

- The proposed model demonstrates the highest median Dice coefficient with a narrow interquartile range, indicating both superior and consistent segmentation performance.
- EfficientNet-B7 and FPN-Seresnet50 also achieve high median Dice coefficients but exhibit slightly larger variability compared to the proposed model.
- Models like FR2PAttU-Net and Hybrid V-Net show lower median values with wider spreads, suggesting greater inconsistency in their segmentation results.

Unique architectural elements or focused preprocessing and postprocessing actions, including improved contrast adjustments or morphological procedures, which sharpen boundaries and cut down on noise, are probably the cause of this improvement. In medical imaging, where segmentation accuracy has a direct impact on therapeutic and diagnostic choices, the high precision attained by the suggested approach is very important. As a result, our model’s performance indicates that it might be used as a standard for kidney image segmentation, providing a reliable choice for critical medical applications where accuracy is crucial.

## 5. Limitations and Future Works

While the proposed approach demonstrates significant advancements in kidney segmentation, this study has certain limitations that should be addressed in future research:

- The model was primarily trained and evaluated on the KiTS19 dataset, which may not fully represent the diversity of real-world clinical imaging protocols and patient populations. Validation of additional datasets from varied sources is needed to ensure generalizability.
- The modified CLAHE preprocessing algorithm relies on fine-tuned parameters (NT and CL) that may need to be adjusted for other datasets. This parameter sensitivity could limit its applicability in scenarios where diverse image characteristics are encountered.
- The preprocessing steps, including the modified CLAHE algorithm, and the CNN architecture require significant computational resources, which may limit applicability in resource-constrained settings.
- The study focuses exclusively on kidney segmentation. Extending the methodology to other organ segmentation tasks would provide a more comprehensive evaluation of its applicability.

Potential future research directions include:

- Extending the evaluation of the proposed model to datasets beyond KiTS19, including images from different imaging modalities (e.g., MRI, ultrasound) and clinical settings, to ensure broader applicability and robustness.
- Adapting the proposed preprocessing techniques and segmentation model to other organ segmentation tasks, such as liver, pancreas, or tumor segmentation, to explore its versatility.
- Incorporating the segmentation model into multi-task frameworks that can simultaneously perform detection, classification, and segmentation for comprehensive medical image analysis.
- Exploring adaptive preprocessing algorithms that can automatically adjust parameters like NT and CL based on image characteristics to eliminate the need for manual tuning.

We plan to use our approach to study uncertainty estimations in future work, as they are important for medical image segmentation for tumor processing. We are also thinking about adding transformers to our model because current computer vision research has shown that this architecture significantly improves state-of-the-art segmentation results. Furthermore, data augmentation receives too little attention. A lack of data is always affecting model performance in the field of medical image analysis since it is hard to obtain and label data [51,52,53,54,55,56,57,58]. A significant area of research to address this issue is the data augmentation method, even though it has not received much attention in previous studies. There are only a few recently suggested data augmentations and a few standard data augmentations, such as the GAN-based approach. Consideration should be given to more complex and sophisticated data augmentation techniques [59,60,61,62,63].

## 6. Conclusions

This study explored the effectiveness of various preprocessing techniques on improving the segmentation accuracy of CNN models applied to the KiTS19 dataset. Using CLAHE and a modified CLAHE method, we enhanced the dataset’s image quality, as verified by BRISQUE scores, aiming to improve the clarity and detail necessary for accurate segmentation. By systematically refining the image quality, our approach provided CNN with optimized data inputs for learning detailed kidney features.

Our experiments indicated that higher BRISQUE scores correlated with lower segmentation accuracy, emphasizing the influence of image quality. The original KiTS19 dataset, with a BRISQUE value of 28.8, yielded an accuracy of 0.951. Applying CLAHE filtering improved the BRISQUE to 26.4 and raised the model’s accuracy to 0.969. The modified CLAHE method showed the most improvements, lowering the BRISQUE to 21.1 and increasing the model’s accuracy to a remarkable 0.996. These findings demonstrate the potency of sophisticated preprocessing, as the CNN model was able to acquire crucial structural subtleties and achieve very accurate segmentation through meticulous contrast changes made using modified CLAHE. With an IoU of 0.991 and a Dice coefficient of 99.6%, our model surpasses earlier designs, according to experimental results on the KiTS19 dataset, setting new standards for kidney segmentation accuracy. Our model performs better across Dice, precision, sensitivity, and recall when compared to other top models, such as EfficientNet, FPN, and UNet variations. This suggests that our preprocessing and postprocessing methods, along with a precisely calibrated CNN architecture, significantly improve segmentation precision.

In conclusion, our study shows that by improving picture quality and detail, modified CLAHE preprocessing significantly improves CNN segmentation performance. Particularly useful in medical imaging tasks such as kidney segmentation, this preprocessing method raises the possibility that customized preprocessing may be used to additional medical applications that call for high-precision segmentation.

## Figures and Tables

**Table 1:** CLAHE algorithm parameters and BRISQUE indicator values.

NL	CL	BRISQUE
2	0.01	4.573
4	0.01	5.857
6	0.01	5.646
…	…	…
24	1	4.166

**Table 2:** Parameters of collection elements.

Image	NL	CL
X _0_	2	0.02
X _1_	2	0.03
X _2_	4	0.01
…	…	…
X _n_	6	0.04

**Table 3:** Performance comparison of models.

Reference	Method	Dice Coefficient (%)	Precision (%)	Sensitivity (%)	Recall (%)	Dataset
Hsiao et al., 2022 [37]	EfficientNetB5	96.90	97.47	-	96.45	KiTS19
Da Cruz et al., 2020 [38]	UNet2d	96.33	-	95.32	-	KiTS19
Zhao et al., 2020 [39]	UNet3d	96.90	97.10	-	96.80	KiTS19
Li et al., 2022 [40]	ResUnet	96.54	-	96.49	-	Own
Haghighi et al., 2018 [41]	UNet3d	87.50	92.7	-	-	DCE-MRI
UNet2d [42]	UNet2d	96.50	96.55	95.90	96.20	KiTS19
UNet3d [42]	UNet3d	96.80	96.85	96.10	96.25	KiTS19
Cihan Akyel, 2023 [43]	LinkNet	96.62	96.58	96.97	96.18	KiTS19
Cihan Akyel, 2023 [43]	LinkNetB7	97.20	97.30	97	97	KiTS19
Ensemble CNN [44]	Ensemble CNN	85	91	-	87	KiTS19
Proposed model	CNN	99.6	98.7	99.3	98.6	KiTS19

**Table 4:** BRISQUE values of datasets.

Dataset	BRISQUE Value	Neural Network	Accuracy
Original KiTS19	28.8	CNN trained on original KiTS19	0.951
KiTS19 filtered by CLAHE	26.4	CLAHE-based CNN	0.969
KiTS19 filtered by modified CLAHE	21.1	Modified CLAHE-based CNN	0.996

**Table 5:** Comparison metrics of models.

Reference	Model	Dice Coefficient	IoU Scores
[45]	nnU-Net	0.969	-
	Hybrid V-Net	0.962	-
	U-Net3+	0.959	-
	SRE Net 2D-CNN	0.979	-
[46]	FR2PAttU-Net	0.948	-
[47]	U-Net EfficientNet-B0	0.984	0.969
	U-Net EfficientNet-B1	0.981	0.963
	U-Net EfficientNet-B2	0.983	0.966
	U-Net EfficientNet-B3	0.985	0.970
	U-Net EfficientNet-B4	0.987	0.974
	U-Net EfficientNet-B5	0.986	0.972
	U-Net EfficientNet-B6	0.985	0.971
	U-Net EfficientNet-B7	0.988	0.977
[48]	FPN- Seresnet18	0.986	0.972
	FPN- Seresnet34	0.986	0.972
	FPN- Seresnet50	0.990	0.980
	FPN- Seresnet101	0.988	0.976
	FPN- Seresnet152	0.989	0.979
	FPN- Resnet50	0.987	0.974
Proposed model	CNN	0.996	0.991

## Data Availability

The data are contained within the article.
